# *Galleria mellonella* (Lepidoptera: Pyralidae) Hemocytes Release Extracellular Traps That Confer Protection Against Bacterial Infection in the Hemocoel

**DOI:** 10.1093/jisesa/ieab092

**Published:** 2021-12-04

**Authors:** Robin Y Chen, B Andrew Keddie

**Affiliations:** Department of Biological Sciences, University of Alberta, Edmonton, AB T6G 2E9, Canada

**Keywords:** insect immunity, extracellular trap, *Galleria mellonella*, hemocyte

## Abstract

Extracellular traps (ETs) released from vertebrate and invertebrate immune cells consist of chromatin and toxic granule contents that are capable of immobilizing and killing microbes. This recently described innate immune response is not well documented in insects. The present study found that ETs were released by hemocytes of *Galleria mellonella* (Linnaeus) (Lepidoptera: Pyralidae) in vivo and ex vivo after bacterial stimulation. ET release (ETosis), hemolymph coagulation, and melanization likely contributed to the immobilization and killing of the bacteria. The injection of *G. mellonella* hemocyte deoxyribonucleic acid (DNA) in the presence of bacteria increased bacterial clearance rate and prolonged insect survival. Taken together, these results indicate the presence of insect hemocyte extracellular traps (IHETs) that protect the insect against microbial infection in the hemocoel and represent the first documentation of ETs in insects in vivo.

## Graphical Abstract

**Figure:**
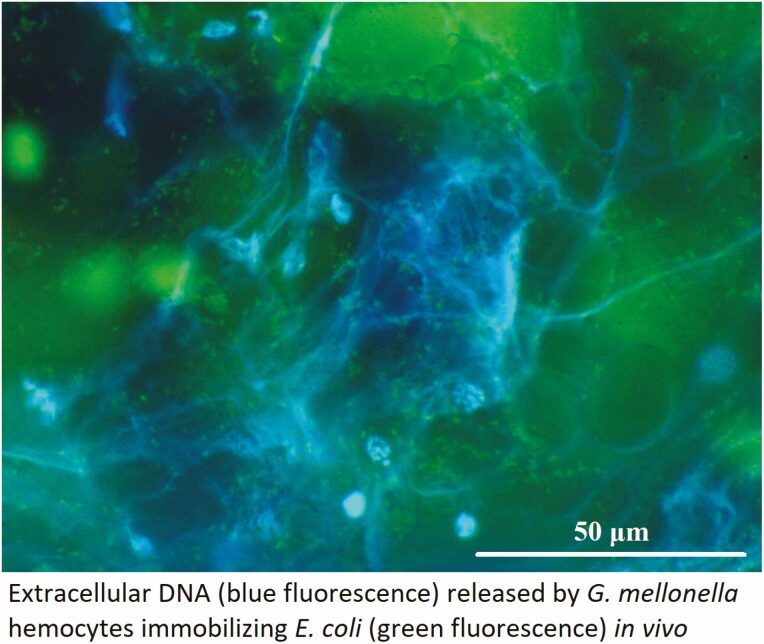

Neutrophil extracellular traps (NETs), first discovered in 2004, are extracellular networks of deoxyribonucleic acid (DNA), histones, and granule contents released by neutrophils that trap and kill microbes including bacteria and fungi (Brinkmann et al. 2004, Urban et al., 2006). The process of NET release is known as NETosis and is considered to be an active form of neutrophil death distinct from apoptosis and necrosis (Brinkmann 2018). The immobilization of microbes by NETs prevents microbial dispersion to other locations within the host. The antimicrobial activity of NETs is attributed to the toxicity of neutrophil granule contents (e.g., neutrophil elastase, myeloperoxidase, lysozyme, and defensins), histones, and DNA (Brinkmann 2018). The two known forms of NETosis result in either the lysis of neutrophils after NET release (suicidal NETosis) or the formation of anuclear neutrophils that remain intact and functional (vital NETosis) (Yipp and Kubes 2013). Suicidal NETosis can be induced by pathogens through cell surface receptors (e.g., Toll-like receptors, Fc receptors, and complement receptors). Receptor activation stimulates the release of Ca^2+^ from the endoplasmic reticulum into the cytoplasm. Increased cytoplasmic Ca^2+^ levels activate protein kinase C (PKC) and result in downstream assembly and activation of the NADPH (nicotinamide adenine dinucleotide phosphate) oxidase complex and the production of reactive oxygen species (ROS). The ROS rupture cytoplasmic granules and the nuclear envelope, resulting in the mixture of granule contents with the nucleoplasm. The granule enzymes neutrophil elastase, myeloperoxidase, and peptidyl-arginine deiminase 4 enter the nucleus and collectively induce chromatin decondensation. Finally, the cell membrane ruptures and release NETs into the extracellular space (Brinkmann 2018). Suicidal NETosis can also be induced chemically by the PKC activator phorbol 12-myristate 13-acetate (PMA) or microbial surface components (e.g., lipopolysaccharides) (Brinkmann et al. 2004). The biochemical mechanisms behind vital NETosis remain unclear to date. NETs are released by exocytosis through the budding of vesicles that transport DNA from the nucleus to the cell membrane. The process is ROS-independent and occurs more rapidly than suicidal NETosis. Neutrophils that have undergone vital NETosis maintain cell membrane integrity and retain the abilities of adhesion, chemotaxis, degranulation, and phagocytosis (Pilsczek et al. 2010). A different form of vital NETosis involving the release of mitochondrial DNA instead of nuclear DNA has also been documented (Yousefi et al. 2009).

In addition to human neutrophils, the release of extracellular traps (ETosis) has also been observed in other vertebrates (e.g., avian heterophils and fish neutrophils), invertebrates (e.g., annelid coelomocytes, cnidarian mesogleal cells, and crustacean hemocytes), and even in plants (root border cells) (Palić et al. 2007, Chuammitri et al. 2009, Hawes et al. 2012, Ng et al. 2013, Robb et al. 2014, Homa 2018). As such, ETosis is considered to be an ancient and evolutionarily conserved immune response in animals (Robb et al. 2014). However, evidence of ETosis in insects, the most diverse group of animals, remains ambiguous. A study reported extracellular DNA release by *Periplaneta americana* hemocytes ex vivo after stimulation with delipidated lipopolysaccharide (LPS) and DH5α *Escherichia coli* (Migula) (Enterobacterales: Enterobacteriaceae) (Nascimento et al. 2018). However, the study lacked controls (i.e., blank stimulation) to account for potential experimental artifacts (e.g., mechanical lysis of hemocytes during handling and spontaneous DNA release) and the hemocyte type(s) involved remain unknown. A more recent study showed that *Rhodnius prolixus* hemocytes of unknown type released extracellular DNA ex vivo after stimulation with LPS and *Staphylococcus aureus* (Grahl et al. 2020). However, the injection of genomic DNA in vivo did not affect the replication of *S. aureus* in the hemolymph, hemocyte aggregation, or melanization. An earlier study found that *Galleria mellonella* L. (Lepidoptera: Pyralidae) oenocytoids lyse rapidly ex vivo, which proposed these hemocytes as a source of extracellular DNA in vivo. The same study also found that extracellular ribonucleic acid (RNA) enhances antimicrobial peptide (AMP) expression and cellular immune response in *G. mellonella* (in the presence of heat-killed bacteria) and extends insect survival time postinfection with the entomopathogen *Photorhabdus luminescens* (Altincicek et al. 2008). The authors claimed that similar results were found when extracellular DNA was used instead of RNA. However, no data was presented to support this claim. To date, no conclusive evidence of the presence of ETs and their effects were documented in insects in vivo.

The present study aims to determine whether hemocytes of *G. mellonella* release ETs, and if so, whether ETs confer protection against bacterial infection in the hemocoel. We found that *G. mellonella* hemocytes release ETs in vivo after intrahemocoelic injection of bacteria and ex vivo after stimulation with bacteria. The injection of *G. mellonella* hemocyte DNA increased bacterial clearance rate and prolonged insect survival. Novel aspects of this research include the documentation of ETs in an insect in vivo for the first time and detailed descriptions of ETs observed ex vivo. These results support the hypothesis that insect hemocytes release extracellular traps that protect the insect against microbial infection within the hemocoel and enable the use of *G. mellonella* as a novel model organism for the study of ETs.

## Materials and Methods

### Insects and Bacteria

*Galleria mellonella* larvae were purchased from Recorp Inc. (Georgetown, Ontario, Canada) and used to establish a laboratory colony. Insects were reared in 20 oz Atlas mason jars kept in a Percival I-41VL incubator at 30°C, 30% RH, and a photoperiod of 0:24 (L:D) h. The larvae were fed ad libitum on artificial diet (Supp Appendix A [online only]). Last instar larvae approximately 300 mg in mass (measured by Mettler College150 digital precision balance) were used for all experiments.

Wild type enteropathogenic *Escherichia coli* (E2348/69 serotype O127:H6, henceforth referred to as simply EPEC) was obtained from T. L. Raivio (University of Alberta). EPEC was transformed with the plasmid pXG-1, enabling the constitutive expression of green fluorescent protein (GFP) for in situ visualization by fluorescence microscopy and chloramphenicol resistance for isolation by selective media (Urban and Vogel 2007). This bacterium was used since 1) EPEC is virulent in the hemocoel of *G. mellonella* (Leuko and Raivio 2012, Chen and Keddie 2021) and 2) extracellular DNA was serendipitously discovered in the hemolymph of *G. mellonella* larvae injected intrahemocoelically with EPEC in a preliminary experiment (personal observation), making EPEC a suitable pathogen to study ETs and their effects in *G. mellonella*. EPEC was cultured in Luria-Bertani (LB) medium (Supp Appendix B.3 [online only]) in glass culture tubes (KIMAX, 16 mm × 100 mm) on a shaker (Mistral Multi-Mixer Model 4600, Lab-Line) at 30°C to match *G. mellonella* rearing temperature. Bacteria were quantified by optical density (OD_600_) using a Spectronic 20+ spectrophotometer preinjection and by the plate-count method using LB agar postinjection (Supp Appendix B.3 [online only]). Log phase bacteria washed and suspended in insect Ringer’s solution (Supp Appendix B.1 [online only], henceforth referred to as simply Ringer’s) were used as inoculum for experiments.

### Insect Injections

A 1 mL glass tuberculin syringe (BD Yale) with a 33-gauge beveled needle mounted on a motorized microapplicator (Model M, ISCO Inc.; Lincoln, NE) was used to inject 5 µL of inoculum into the hemocoel (intrahemocoelic) through the plantar of the left anteriormost proleg of the larva. Injection sites were disinfected by swabbing with 70% ethanol immediately prior to injection. Injections were conducted under a stereo microscope at 12× magnification. Injection sites were selected to minimize bleeding and underlying tissue damage. Larvae were incubated at 30°C postinjection and allowed to feed ad libitum on artificial diet postinjection for the remainder of the experiment.

### Extracellular Traps In Vivo

EPEC (1.5 × 10^4^ CFU) was injected intrahemocoelically into *G. mellonella* larvae as previously described. Larvae injected with Ringer’s were used as control. Before dissection, larvae (25 injected with EPEC, 14 injected with Ringer’s) were surface-sterilized (by sequential immersion: 30s in 70% ethanol → 10s in sterile water → 60s in 10% bleach → 10s in sterile water). These larvae were submerged in Ringer’s and dissected at 24 h postinjection under a stereo microscope using micro scissors. A longitudinal incision was made on the dorsal cuticle of the larvae from the first thoracic segment to the last abdominal segment, followed by two perpendicular lateral incisions on each end of the longitudinal incision, making an ‘I’ shape. The incisions were made very carefully to minimize damage to the underlying tissue. Following the incisions, the insect cuticle was peeled back and pinned to either side, revealing internal structures. Melanized coagula were observed attached to tissue surfaces when present and were photographed in situ (Ricoh R10), then carefully removed from dissected insects using fine tip forceps (Dumont No.5), immersed in Ringer’s, and stained with 10 µg/mL Hoechst 33342. Hemolymph and coagula were visualized by differential interference contrast (DIC) and fluorescence microscopy using a Reichert-Jung Polyvar microscope at 500× magnification and photographed using an Olympus E-420 digital camera.

### Extracellular Traps Ex Vivo

Hemolymph from 2 healthy, untreated *G. mellonella* larvae was collected aseptically in ice-cold anticoagulant antimelanization solution (Supp Appendix B.2 [online only]). Hemolymph was collected by micropipette after creating a small incision at the base of the right anteriormost proleg with micro scissors and the application of gentle pressure to the insect until a droplet of hemolymph appears. Larvae were surface-sterilized by sequential immersion as described previously, immediately prior to hemolymph collection. Hemolymph collection was performed aseptically in a biological safety cabinet (Model 1106, Forma). Hemolymph plasma was removed by micropipette after centrifugation (Eppendorf 5415L) at 200 g for 5 min. The hemocyte pellets were washed once with ice-cold Ringer’s and resuspended in Grace’s insect medium (Supp Appendix B.4 [online only]) by gentle agitation. The hemocytes were loaded onto 6 sterile glass slides with approximately 1.5 × 10^5^ hemocytes per slide. The hemocytes were allowed to adhere to the slides for 30 minutes and were stimulated for 1 h with EPEC (5.0 × 10^4^ CFU), PMA (50 µM), or Ringer’s as control. The hemocytes were subsequently washed with Ringer’s, fixed with 4% formaldehyde for 1 h, and stained with 10 µg/mL Hoechst 33342. The slides were kept at 30°C in sterile glass petri dishes (PYREX, 90 mm diameter) humidified with wet sterile filter paper during all waiting steps to minimize evaporation and contamination. Hemocytes were examined by DIC and fluorescence microscopy using a Reichert-Jung Polyvar microscope at 500× magnification. Hemocytes releasing extracellular DNA and the total number of hemocytes were quantified in 5 random fields of views for each slide and were photographed using an Olympus E-420 digital camera. The hemocyte types responsible for DNA release were identified morphologically when possible based on the descriptions of Wu et al. (2016).

### Addition of Extracellular DNA In Vivo

*G. mellonella* larvae were injected intrahemocoelically with two doses of EPEC (1.6 × 10^4^ CFU or 2.2 × 10^4^ CFU) and a Ringer’s control in the presence or absence of *G. mellonella* hemocyte DNA (500 ng, dissolved and suspended in Ringer’s) (Supp Appendix C [online only]). These doses were selected based on an extensive (*n* = 349) LD_50_ experiment using 18 different EPEC doses spanning 0–4.0 × 10^4^ CFU (Chen and Keddie 2021). Doses between 8.4 × 10^3^ CFU and 2.9 × 10^4^ CFU result in all disease outcomes (i.e., recovery and mortality present in different insects at the same dose). The EPEC doses in the current experiment were selected in order to examine the impact of DNA injection on both lethal and sublethal effects of EPEC in *G. mellonella*. Larvae injected with Ringer’s or DNA in the absence of EPEC were used as controls. A total of 240 insects (across 6 treatments) were used in this experiment with at least 30 insects per treatment (Supp Appendix C [online only]). Hemolymph was collected at 1 h, 3 h, 6 h, 24 h, and 48 h postinjection aseptically from 4 to 8 insects per treatment per time point without replacement to quantify circulating EPEC by plate-count using LB agar. The remaining insects (10 or 20 of each treatment) were left undisturbed to obtain mortality, time to pupation, and survival score. Insect mortality was recorded daily until day 20 postinjection at which point all insects had either died or emerged as adults. Larvae were considered dead when no movement was observed after tactile stimulation. Survival scores were calculated for each insect:


$$\begin{array}{l} Survival\;Score=\;\frac{Survival\;Time}{20} \end{array}$$

which were used as proxies for survival time (days) in the analysis to avoid heteroscedasticity. Time to pupation was recorded as the number of days postinjection until pupation. The DNA used in this experiment was extracted from hemocytes of untreated *G. mellonella* larvae using a DNeasy Blood and Tissue Kit (Qiagen, Germantown, MD). DNA concentration was quantified by a Qubit fluorometer (Invitrogen, Waltham, MA) and DNA purity was assessed with a NanoDrop spectrophotometer (Thermo, Waltham, MA). DNA was pelleted and dried by a centrifugal evaporator (DyNA Vap, Labnet, Edison, NJ) and redissolved in Ringer’s to reach the appropriate final concentration (verified by a Qubit fluorometer).

### Statistical Analyses

Statistical analyses were conducted using R (R Core Team 2019). Generalized linear models (GLMs) were constructed to determine the effects of the addition of extracellular DNA on EPEC clearance, insect mortality, survival score, and time to pupation. The GLM family used in each model was determined by the type of data: Gaussian for continuous data such as pupal mass, Poisson for count data such as time to pupation, and binomial for proportion data such as mortality. Overdispersion and underdispersion were accounted for by using quasi-families. Model assumptions were checked graphically. The minimum adequate models were obtained by stepwise deletion of nonsignificant factors and interactions when applicable. Tukey contrasts (pairwise comparisons) were used to determine where significant differences occurred post hoc to GLMs. *P*-values were adjusted to account for multiple comparisons.

## Results

### Extracellular Traps In Vivo

**Fig. 1.**
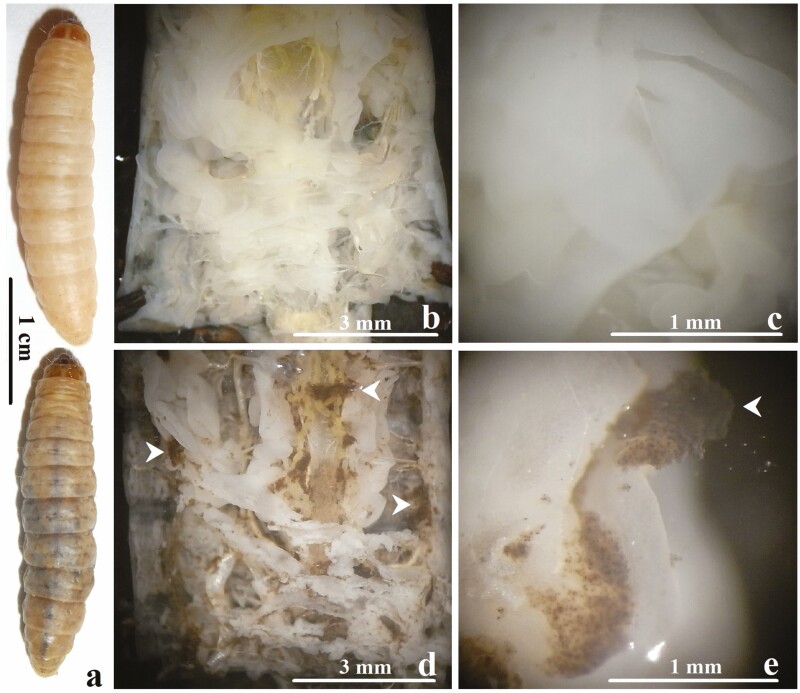
*G. mellonella* larvae at 24 h after intrahemocoelic injection with Ringer’s (top) and 1.5 × 10^4^ EPEC (bottom). (a) Cuticular melanization was observed in larvae injected with EPEC but was absent in control larvae. (b–e) Upon dissection, melanized coagula (arrowheads) were seen attached to surfaces of various tissues and organs (e.g., fat body) in the hemocoel of diseased larvae (d, e) but were absent in control larvae (b, c).

**Fig. 2.**
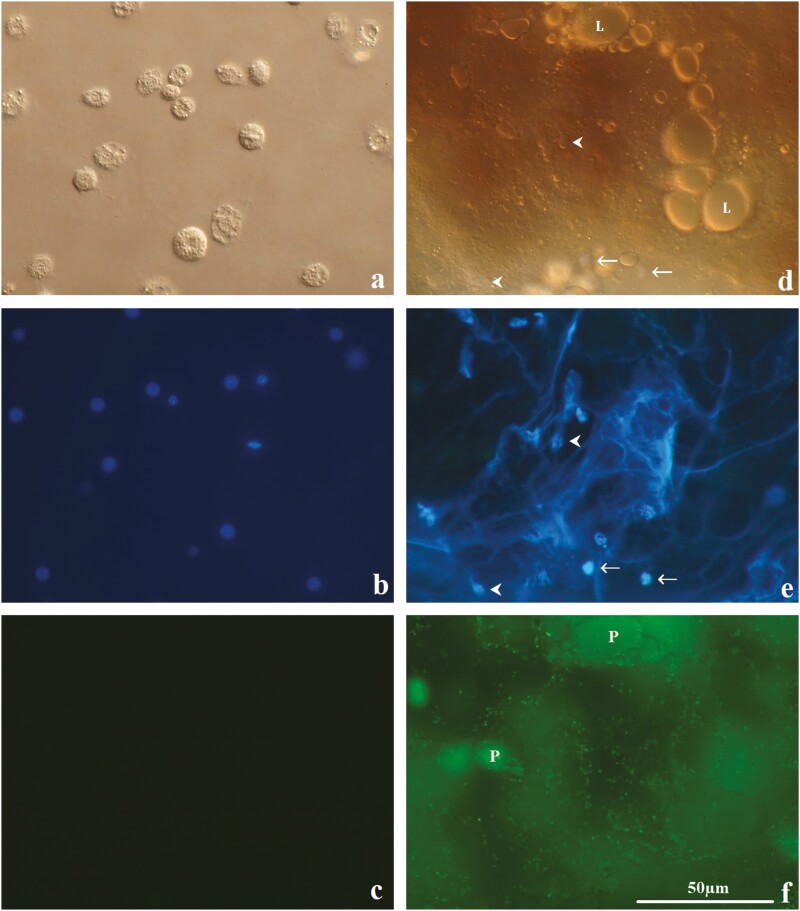
(a–c) Hemocytes from a *G. mellonella* larva at 24 h postinjection with Ringer’s displaying: (a) typical hemocyte morphology, (b) typical hemocyte nuclei (blue fluorescence by Hoechst 33342 staining), and (c) absence of green fluorescence. (d–f) Melanized coagulum from the surface of the fat body of a *G. mellonella* larva at 24 h postinjection with 1.5 × 10^4^ EPEC visualized with DIC and fluorescence microscopy. Both sets of images were taken at the same magnification, position, and focal plane. (d) Lipid droplets or gas pockets (‘L’) and hemocytes (arrows) were embedded in the coagulum. (e) DNA was seen projecting from some of the hemocytes (arrowheads) to form a net-like structure. (f) The network of DNA was co-localized with EPEC expressing GFP. Dense pockets of trapped EPEC (‘P’) were found on different focal planes within the coagulum.

Dissection of larvae at 24 h postinjection with EPEC (Fig. 1a, bottom) revealed melanized coagula in the hemocoel adhering to surfaces of various tissues and organs including fat body, trachea, and gut (Fig. 1d and e) and were absent in control larvae (Fig. 1a, top; Fig. 1b and c). Extracellular DNA was found within melanized coagula in 40% of EPEC-injected insects (Fig. 2d and e) and was absent in control larvae (Fig. 2a and b). Extracellular DNA appeared as intricate, irregularly-shaped networks of intense blue fluorescence after Hoechst staining and were highly variable in size. EPEC was associated with 90% of the extracellular DNA observed (Fig. 2f) and was absent in control larvae (Fig. 2c). Extracellular DNA was observed in the hemolymph as early as 1 h postinjection (personal observation).

### Extracellular Traps Ex Vivo

**Fig. 3.**
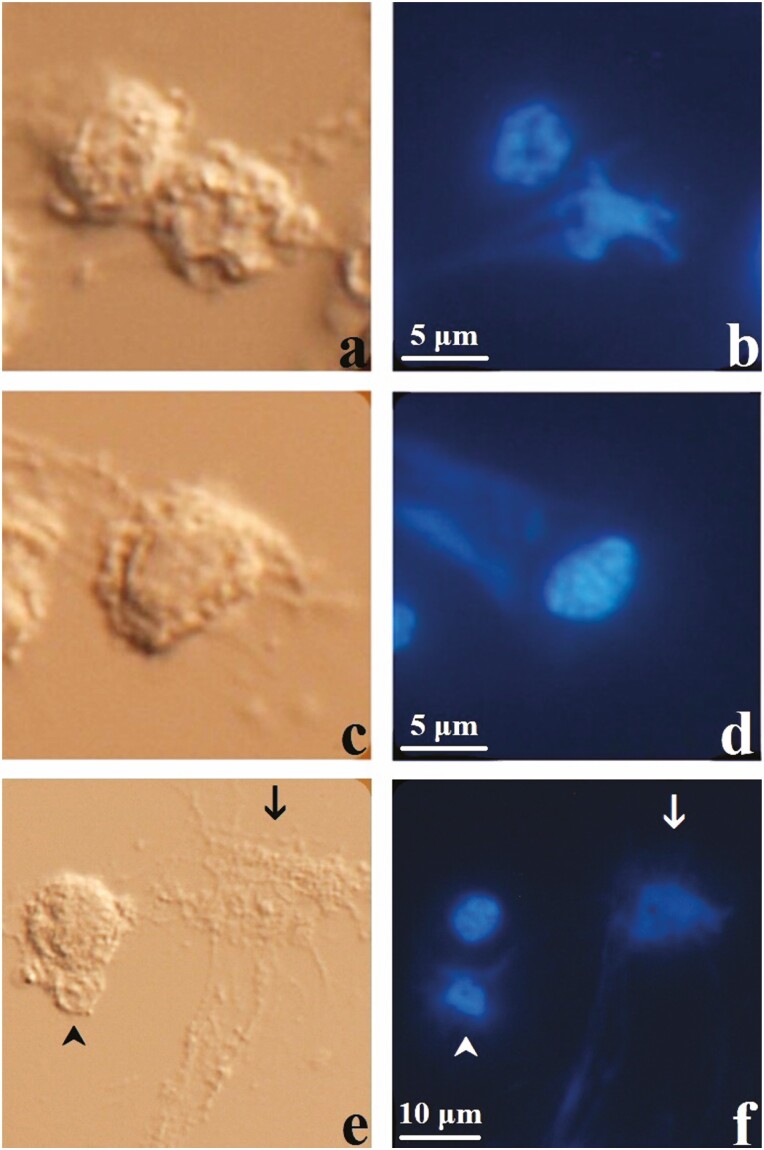
*G. mellonella* hemocytes stimulated for 1 h with EPEC (5.0 × 10^4^ CFU), stained with Hoechst 33342, and visualized with DIC (left column) and fluorescence microscopy (right column). (a, b) Extracellular DNA released by a granulocyte with irregular-shaped nucleus with diffuse staining compared to adjacent hemocytes. (c, d) Extracellular DNA released by a granulocyte with intact nuclear envelope and cell membrane. The extracellular DNA appeared to have originated from the cytoplasm with fibrillar extracellular projections. (e, f) Extracellular DNA released by a lysed hemocyte (arrow) and an exposed hemocyte nucleus (arrowhead).

Extracellular DNA release was observed from 0.20% (12 out of 5,977) of hemocytes stimulated with EPEC, 0.08% (5 out of 6,572) of hemocytes stimulated with PMA, and 0.02% (1 out of 4,562) of the hemocytes stimulated with Ringer’s. EPEC stimulation resulted in significantly higher proportion of hemocytes releasing extracellular DNA compared to control hemocytes stimulated with Ringer’s (χ ^2^ = 5.3, df = 1, *P* = 0.02). The proportion of hemocytes releasing extracellular traps after PMA stimulation was also higher compared to the control hemocytes stimulated with Ringer’s, but this difference was not statistically significant (χ ^2^ = 0.63, df = 1, *P* = 0.42). Three patterns of DNA release were observed: Pattern I (Fig. 3a and b, and Supp Fig. 1 [online only]): the hemocyte produced fibrillar projections of DNA that originated from the nucleus. Nucleus appeared irregular in shape with diffuse fluorescence compared to normal nuclei. The hemocyte appeared relatively intact. Pattern II (Fig. 3c and d): the hemocyte produced fibrillar projections of DNA that appeared to originate from the cytoplasm. Nucleus appeared normal and the hemocyte appeared intact. Pattern III (Fig. 3e and 3f, and Supp Fig. 2 [online only]): the hemocyte lysed and the nucleus appeared diffuse or irregular in shape, with fibrillar DNA projecting from the naked nucleus into the extracellular space. Hemocytes exhibiting patterns I and II of DNA release were tentatively identified as granulocytes due to the presence of numerous cytoplasmic granules. Lysed hemocytes were identified as granulocytes only when the exposed cytoplasm contained numerous granules, and those with few or no cytoplasmic granules could not be identified. No hemocyte lysis was observed after stimulation with Ringer’s. The granulocyte that released DNA after stimulation with Ringer’s was classified as pattern I. The hemocytes stimulated with EPEC displayed all three patterns of DNA release (patterns I – III from granulocytes and pattern III of unidentified hemocyte) whereas only pattern III was found in the hemocytes stimulated with PMA. No EPEC was found to be associated with extracellular DNA. In general, hemocytes stimulated with PMA appeared more flattened compared to hemocytes stimulated with Ringer’s or EPEC. Cellular debris was more abundant in the background of hemocytes stimulated with PMA or EPEC compared to the backgrounds of hemocytes stimulated with Ringer’s. Results of this experiment were summarized in Table 1.

**Table 1.** DNA release from *G. mellonella* hemocytes ex vivo

Treatment	Proportion of hemocytes releasing DNA	n	*P*-value*	Types of hemocytes releasing DNA	Patterns of DNA release
Ringer’s (control)	0.02%	4562	n/a	Granulocyte	I
EPEC (5.0 × 10^4^ CFU)	0.20%	5977	0.02	Granulocytes and unidentified hemocytes	I, II, III
PMA (50 μM)	0.08%	6572	0.42	Unidentified hemocytes	III

*P-values were calculated using χ ^2^ tests for equality of proportions against the control.

### Addition of Extracellular DNA In Vivo

**Fig. 4.**
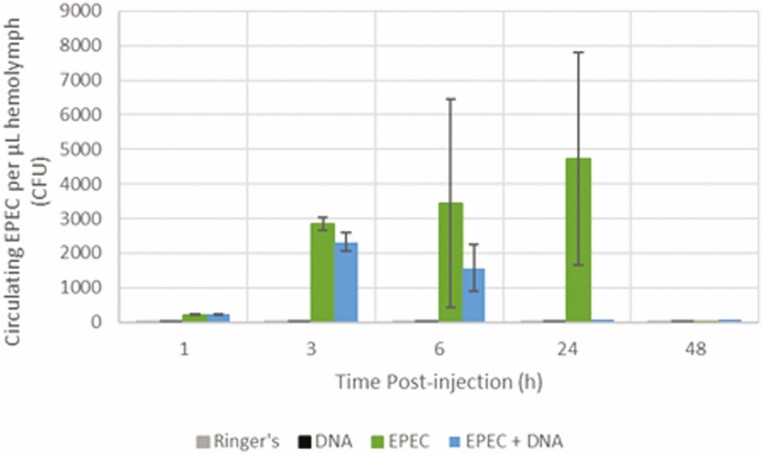
Average number of circulating EPEC (± SE) per µL hemolymph at various time points post-injection in *G. mellonella* larvae injected intrahemocoelically with EPEC only (10^4^ CFU), EPEC (10^4^ CFU), and 500 ng of DNA, Ringer’s, or DNA only (*n* = 160). EPEC was cleared faster and earlier in larvae injected with EPEC and DNA (24 h postinjection) compared to larvae injected with EPEC alone (48 h postinjection).

**Fig. 5.**
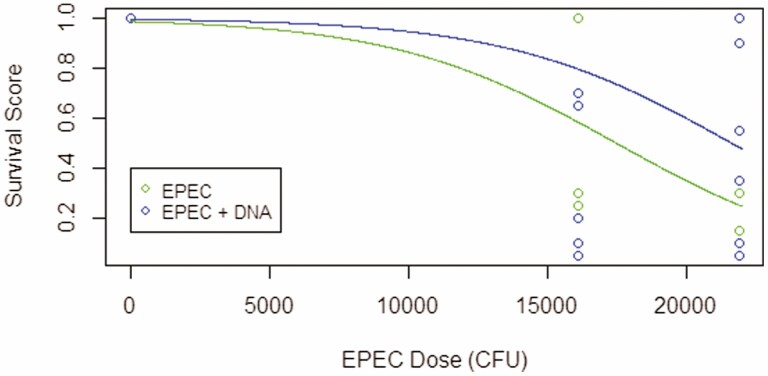
Survival score of *G. mellonella* larvae injected intrahemocoelically with EPEC only (10^4^ CFU) and larvae injected with EPEC (10^4^ CFU) and 500 ng of DNA (*n* = 80). Insects injected with EPEC and DNA showed higher survival scores (i.e., survived approximately 1 d longer on average) compared to insects injected with EPEC alone.

DNA injection (Quasi-Poisson GLM, F = 11.9, df = 1, 157, *P* = 0.0007), time postinjection (F = 11.1, df = 4, 153, *P* < 0.0001), and EPEC dose (F = 67.8, df = 1, 158, *P* < 0.0001) were all significant predictors of circulating EPEC count. Statistically significant differences in circulating EPEC count among treatments over time were detected (F = 3.15, df = 4, 149, *P* = 0.02). Overall, larvae injected with EPEC and DNA showed reduced number of circulating EPEC and earlier clearance compared to larvae injected with only EPEC (Fig. 4). EPEC dose was positively associated with the number of circulating EPEC (β = 1.65 × 10^−4^, *P* < 0.0001). Bacteria were not found in the hemolymph of larvae without EPEC injections in both treatments and at all time points. EPEC dose but not treatment was a significant predictor of insect mortality (Binomial GLM, deviance = 55.5, df = 1, 78, *P* < 0.0001). No mortality was observed in the absence of EPEC (i.e., Ringer’s alone and DNA in Ringer’s). Mortality increased as EPEC dose increased (β = 1.64 × 10^−4^, *P* < 0.0001). Both treatment (Quasi-binomial GLM, F = 6.39, df = 1, 76, *P* = 0.01) and EPEC dose (F = 94.9, df = 1, 76, *P* < 0.0001) were significant predictors of insect survival score. Insects injected with EPEC and DNA survived approximately 1 d longer on average compared to insects injected with only EPEC (Fig. 5). Survival score was negatively associated with EPEC dose (β = −2.47 × 10^−4^, *P* < 0.0001). EPEC dose but not treatment was a significant predictor of time to pupation post-injection (Quasi-Poisson GLM, F = 90.2, df = 1, 51, *P* < 0.0001). Time to pupation was positively associated with EPEC dose (β = 3.25 × 10^−5^, *P* < 0.0001) (Supp Fig. 3 [online only]).

## Discussion

### Release of Extracellular Trap In Vivo

Hoechst 33342 staining revealed extracellular DNA within melanized coagula that immobilized EPEC (Fig. 2d–f). Indicating that *G. mellonella* hemocytes released DNA upon activation by EPEC, since hemocytes were photographed in the process of DNA release while DNA release was not observed in control larvae (Fig. 2b and e). Oenocytoids lyse upon activation to release prophenoloxidase (PPO) and the exposed nuclei may also rupture to release DNA (Altincicek et al. 2008). Granulocytes may lyse upon contact with foreign objects and contribute to DNA release (Pech and Strand 1996). Hemocyte nuclei were embedded within the coagulum, which may include granulocytes and oenocytoids (Fig. 2e). Extracellular DNA appear to have trapped EPEC upon contact and induced hemolymph coagulation that also immobilized EPEC in the surrounding area, limiting EPEC spread in the hemocoel. Extracellular DNA is known to induce hemolymph coagulation in *G. mellonella* (Altincicek et al. 2008). The procoagulant activity of extracellular DNA likely synergizes with the degranulation of granulocytes and the lysis of oenocytoids, inducing hemolymph coagulation and melanization, contributing to the trapping and killing of EPEC, though this remains to be determined. Hemolymph coagulation potentially enhanced EPEC killing by increasing local concentration of ROS and cytotoxic quinones produced by melanization and granule contents (e.g., lysozyme) released by the degranulation of granulocytes (Chain and Anderson 1983, Nappi et al. 1995). A recent study found that *Periplaneta americana* hemocytes may also release DNA ex vivo which immobilize bacteria (Nascimento et al. 2018). However, release of ETs has not previously been documented in vivo in insects to our knowledge and represents a novel form of insect immune response against microbial pathogens in addition to the typical insect antimicrobial immune responses (i.e., melanization, hemolymph coagulation, AMP production, phagocytosis, and nodulation).

### Release of Extracellular DNA Ex Vivo

The release of extracellular DNA from *G. mellonella* hemocytes was confirmed ex vivo, implicating hemocytes in ETosis. All three treatments (i.e., EPEC, PMA, and Ringer’s) resulted in extracellular DNA release. The single granulocyte that released DNA after stimulation with Ringer’s (Supp Fig. 1 [online only]) was unexpected and could have occurred spontaneously or as a result of activation against the un-coated glass slide. Slides coated in materials that mimic *G. mellonella* basal lamina (e.g., collagen IV, laminin, nidogen, and perlecan) could be used in future experiments to minimize undesired hemocyte activation. Hemocyte lysis was not observed after stimulation with Ringer’s, indicating that handling did not result in mechanical damage to the hemocytes. Oenocytoid lysis observed by Altincicek et al. (2008) after hemolymph collection was likely avoided in the present study by the use of ice-cold isotonic solutions and the removal of hemolymph plasma that contain damage signals from the wound created for hemolymph collection. The flattened appearance of the hemocytes after PMA stimulation may be due to increased cell adhesion, a known effect of PMA on neutrophils (Webster et al. 1986). EPEC stimulation increased the proportion of hemocytes releasing DNA compared to the control, indicating that EPEC was an inducer of DNA release. The increase in the proportion of hemocytes after PMA stimulation compared to the control was not statistically significant, indicating that PMA may not be an inducer of DNA release for hemocytes. However, it is possible that PMA requires a longer stimulation time than 1 h to induce DNA release from hemocytes, which requires future experiments to determine. Extracellular DNA was released by *G. mellonella* granulocytes stimulated with EPEC either from the nucleus (pattern I) or seemingly from the cytoplasm (pattern II). The diffuse fluorescence and the irregular shape of the nucleus (pattern I, Fig. 3b) indicate, respectively, the decondensation of chromatin and the loss of nuclear envelope integrity, which are necessary steps of suicidal NETosis. Similar (pattern I) release of extracellular DNA was recently described from *R. prolixus* hemocytes ex vivo (Grahl et al. 2020). The relatively intact appearance of the hemocyte (Fig. 3a) suggests incomplete DNA release at the time of fixation and staining, which could culminate in the loss of cell membrane integrity and the release of the remaining DNA and cytoplasm into the extracellular space. Granulocyte lysis observed by Pech and Strand (1996) may be the end result of DNA release. Suicidal NETosis may take several hours to complete whereas vital NETosis only takes minutes (Yipp and Kubes 2013). Time-lapse microscopy and longer stimulation times in future experiments are required to capture the entire process of DNA release and may result in a larger proportion of DNA-releasing hemocytes. The intact nucleus and cell membrane with seemingly cytoplasmic origin of DNA release (pattern II, Fig. 3c and d) could indicate vital NETosis, in which the release of nuclear DNA by vesicular transport through the cytoplasm and/or the release of mitochondrial DNA are responsible. Anuclear hemocytes were not observed and the presence of condensed chromatin in the nucleus indicate that the release of mitochondrial DNA may be the case. Alternatively, a partial DNA release from the nucleus could also account for this pattern. Future experiments combining the fluorescent staining of DNA and histones could differentiate between nuclear and mitochondrial DNA. The complete lysis of hemocytes (pattern III; Fig. 3e and f, and Supp Fig. 2 [online only]) most closely resembles suicidal NETosis in that the decondensation of chromatin and the rupture of the nuclear envelope and the cell membrane were all present. The absence of granules in the cytoplasmic remains of some of the lysed hemocytes may indicate either suicidal NETosis of granulocytes that have already degranulated or the lysis of oenocytoids, plasmatocytes, or prohemocytes. Fluorescent antibodies specific to each hemocyte type would enhance hemocyte identification where identification by morphology using DIC microscopy alone is insufficient. EPEC was able to induce all three patterns of DNA release whereas only pattern III was observed after PMA stimulation. This was expected since both suicidal (ROS-dependent) and vital (ROS-independent) NETosis are stimulated by microbes whereas PMA activates PKC and only stimulates suicidal NETosis through oxidative burst (Brinkmann et al. 2004, Pilsczek et al. 2010, Yipp and Kubes 2013). Plasmatocytes and granulocytes of *G. mellonella* are capable of oxidative burst mediated by NADPH oxidase homologous to human neutrophils (Bergin et al. 2005). Oenocytoid lysis in *Spodoptera exigua* is mediated by PKC through bacteria-induced eicosanoid signaling (Shrestha and Kim 2009). This provides additional support for the involvement of *G. mellonella* granulocytes and oenocytoids in the release of extracellular DNA. The regulated nature of oenocytoid lysis may in fact be a form of ETosis and synergizes with PPO release and melanization to trap and kill microbes. The presence of exposed nuclei (Fig. 3c and f) indicates the loss of cell membrane integrity before the loss of nuclear envelope integrity, which occurred in the opposite order compared to suicidal NETosis. Similarities and differences between NETosis and hemocyte extracellular DNA release observed in this study were summarized in Table 2.

**Table 2.** Similarities and differences between NETosis and hemocyte extracellular DNA release observed in this study

Cellular events	Suicidal NETosis	Vital NETosis (nuclear)	Vital NETosis (mitochondrial)	Pattern I	Pattern II	Pattern III
Chromatin decondensation	Yes	Yes	Unknown	Yes	No	Yes
Nuclear envelope rupture	Yes	No	No	Yes	No	Yes
Cell lysis	Yes	No	No	No	No	Yes

No EPEC was trapped by the extracellular DNA ex vivo, which was likely due to the small number of DNA-releasing hemocytes that were only able to cover small areas and EPEC escape from the sparse extracellular DNA. A longer incubation time with a larger amount of EPEC could result in more extracellular DNA release and more frequent EPEC contact with the extracellular DNA. The formation of extensive networks of extracellular DNA observed in *G. mellonella* in vivo after intrahemocoelic EPEC injection (Fig. 2e) likely involved the coordinated release of DNA by numerous hemocytes followed by the coagulation of the surrounding hemolymph and the melanization of the coagulum. Coordinated DNA release was not observed ex vivo, possibly due to the removal of hemolymph plasma factors that may be involved and/or due to other factors from the artificial nature of the ex vivo environment such as the use of un-coated glass slides and cell culture medium. This may be responsible for the rarity of DNA release from hemocytes ex vivo. Removal of hemolymph plasma also prevented coagulation that could otherwise enhance EPEC immobilization and structurally reinforce the extracellular DNA. Conversely, the ability of DNA release by hemocytes ex vivo under plasma-free conditions indicates that hemolymph plasma components are not necessary for DNA release. In a preliminary experiment, melanization, hemolymph coagulation, nodulation, but no extracellular DNA release were observed after ex vivo incubation of whole *G. mellonella* hemolymph with EPEC, similar to the lack of DNA release observed by Altincicek et al. (2008) after ex vivo stimulation of hemocytes using bacteria (data not shown). The reason behind this is unknown but may be due to the presence of hemolymph proteins involved in hemocyte regulation and signaling that may have inhibited DNA release ex vivo. The antimicrobial activity of the extracellular DNA is unknown and requires microbial killing assays to establish. The granule contents of insect granulocytes remain poorly characterized to date. Granules of *G. mellonella* granulocytes contain lysosomal enzymes (e.g., lysozyme) but cannot be distinguished from lysosomes (Chain and Anderson 1983). The simultaneous fluorescent staining of DNA and lysozyme could help determine whether granule/lysosomal contents were associated with extracellular DNA released by hemocytes, similar to NETs. Though it is likely that the DNA release in insect granulocytes and NETosis are homologous, the biochemical mechanism in insects remains unknown and require extensive research to characterize. Future experiments that examine histone citrullination and the association of granulocyte granule contents with the extracellular DNA are required to definitively determine whether ETosis or necrosis is the cause of DNA release (Yipp and Kubes 2013). Preliminary experiments revealed: 1) Intrahemocoelic injection of EPEC induced extracellular DNA release in *Bombyx mori* in vivo (Supp Fig. 4 [online only]). 2) Intrahemocoelic injection of *Candida rugosa* induced extracellular DNA release in *G. mellonella* in vivo (Supp Fig. 5 [online only]). 3) Microbial surface components (LPS and β-glucan) induced extracellular DNA release in *G. mellonella* hemocytes ex vivo (Supp Fig. 6 [online only]). These findings, in addition to studies mentioned previously, indicate that extracellular DNA release may be common in insects and can be induced by different types of microbes (e.g., bacteria and fungi) (Altincicek et al. 2008, Nascimento et al. 2018, Grahl et al. 2020). Future studies are required to determine the prevalence and specificity of extracellular DNA release in insects.

In consideration of the multiple potential hemocytic origins and pathways of the release of extracellular DNA, we propose the term insect hemocyte extracellular traps (IHETs) to be used to collectively describe the extracellular traps released by insect hemocytes that immobilize and potentially kill microbes. The naming scheme avoids confusion with heterophil extracellular traps (HETs) and hemocyte extracellular traps from other invertebrates.

### Addition of Extracellular DNA In Vivo

In a previous experiment, the effects of IHETs in vivo against EPEC infection in *G. mellonella* could not be determined by the injection of DNase I due to the inhibition of DNase I endonuclease activity in vivo (data not shown). The present experiment used an alternative approach by the injection of EPEC in the presence or absence of *G. mellonella* hemocyte DNA. Larvae injected with EPEC and DNA cleared EPEC faster and survived longer compared to larvae injected with EPEC alone, indicating that extracellular DNA confers protection to *G. mellonella* against EPEC. The cation chelation property of DNA destabilizes bacterial cell membrane on contact, resulting in the lysis of the bacterium (Halverson et al. 2015). DNA also induces hemolymph coagulation in *G. mellonella* (Altincicek et al. 2008). The amount of injected hemocyte DNA (500 ng) is equivalent to the complete DNA release from approximately 27% of total circulating hemocytes in a last instar *G. mellonella* larva, assuming genome size of 578 Mbp, average base pair mass of 650 Da, diploid hemocytes, and 1.46 × 10^6^ circulating hemocytes per larva (Jones 1967, Lange et al. 2018). The procoagulant and antimicrobial activity of the injected DNA are likely responsible for enhanced trapping and killing of EPEC in *G. mellonella*, resulting in increased EPEC clearance rate and prolonged survival in the larvae injected with EPEC and DNA compared to the larvae injected with only EPEC. However, the injection of DNA did not reduce insect mortality. This may be due to the absence of granule contents that are normally associated with NETs (and potentially associated with IHETs) reducing the efficacy of the injected DNA in trapping and killing EPEC. The injection of pure DNA may severely underrepresent the true antimicrobial capabilities of IHETs despite the increased cation chelation ability of pure DNA compared to chromatin due to the absence of histones.

Insects from the control groups (i.e., larvae injected with Ringer’s or DNA in Ringer’s) did not differ from each other in mortality, survival time, or time to pupation, indicating that DNA is not toxic to *G. mellonella* at the dose of 500 ng/larva. The overall EPEC dose-dependent increase in mortality, decrease in survival time, and delay in pupation indicate that EPEC is virulent in the hemocoel of *G. mellonella*, manifesting as both lethal and sublethal effects. Interestingly, larvae were able to clear EPEC from the hemolymph by 48 h postinjection, yet death still occurred after bacterial clearance (Fig. 4). This phenomenon was observed in a previous study associated with moribund larvae exhibiting anorexia, lethargy, brachytosis, abnormal frass production, and diarrhea that were able to remain alive for up to 20 d postinjection before finally succumbing to death, likely due to irrecoverable damage to the larva by EPEC and the immune responses despite EPEC clearance (Chen and Keddie 2021).

### Conclusions

The pioneering work by Altincicek et al. (2008) revealed protective effects of extracellular RNA (and possibly DNA) in *G. mellonella* during bacterial infection, but was unable to induce hemocyte ETosis ex vivo. The present study provides evidence of *G. mellonella* hemocyte ETosis both in vivo and ex vivo for the first time. This work represents the first known documentation of IHETs in vivo, which likely synergize with hemolymph coagulation and melanization to immobilize and kill microbes. The release of extracellular DNA from granulocytes was induced by EPEC under plasma-free conditions, with features resembling suicidal or vital NETosis and possibly sharing similar pathways. The lysis of oenocytoids may represent a novel form of ETosis unique to insects. However, additional research is needed to confirm hemocyte identity, characterize the processes and mechanisms of IHET release, and determine the antimicrobial activities of IHETs. The injection of hemocyte DNA conferred limited protection to *G. mellonella* against EPEC by increasing EPEC clearance rate and insect survival time but did not reduce insect mortality. However, the injection of DNA likely underrepresented the true antimicrobial effects of IHETs due to the absence of other potential IHET components. Overall, the results from this study indicate that IHETs are released by *G. mellonella* hemocytes and confer protection to the insect against EPEC infection of the hemocoel, supporting the hypothesis that insect hemocytes release extracellular traps as an immune response that protects the insect against microbial infection of the hemocoel and making the *Galleria*-EPEC system a novel model for the study of extracellular traps.

## Supplementary Material

ieab092_suppl_Supplementary_Materials

ieab092_suppl_Supplementary_Appendices
