# Supplementary material for: Galleria mellonella (Lepidoptera: Pyralidae) Hemocytes Release Extracellular Traps That Confer Protection Against Bacterial Infection in the Hemocoel
Source: J Insect Sci. 2021 Dec 4;21(6):17. doi: 10.1093/jisesa/ieab092 (PMC8643984; doi:10.1093/jisesa/ieab092)
Supplement: ieab092_suppl_Supplementary_Materials [file ieab092_suppl_supplementary_materials.docx]

**Supplementary Material**


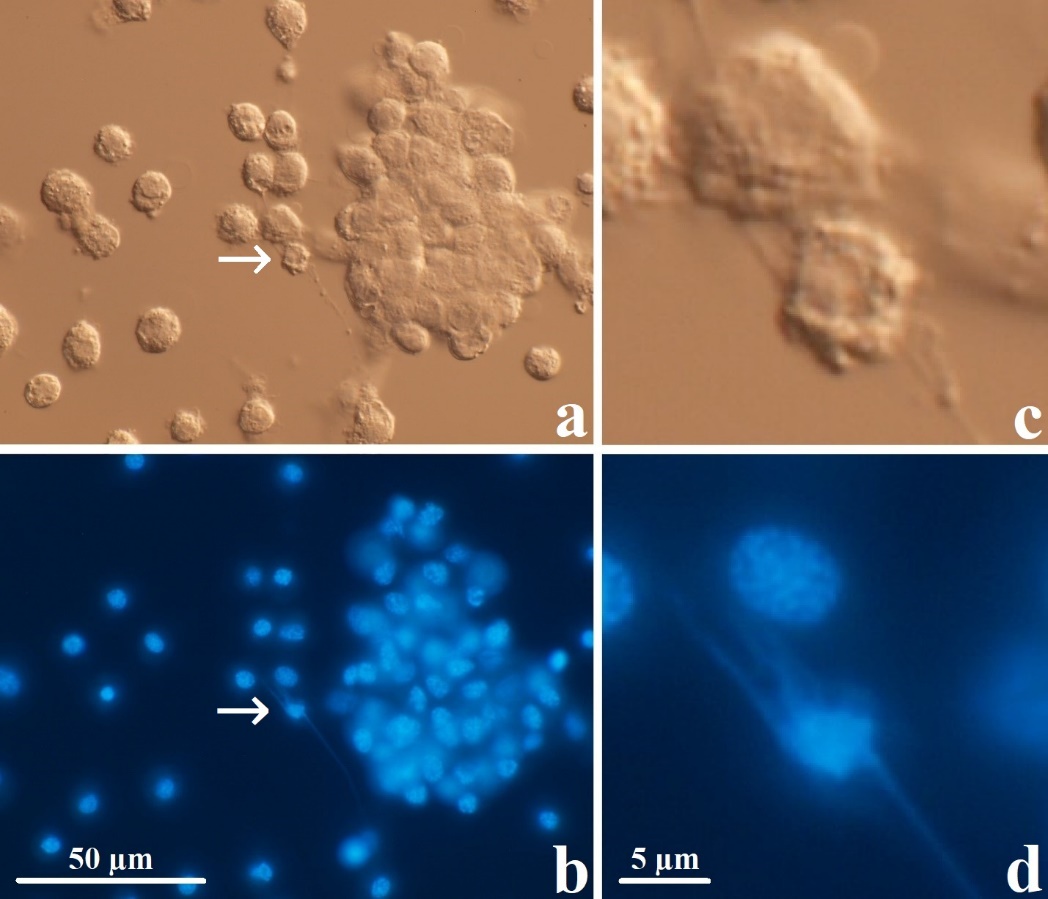


Figure S.1 *G. mellonella* hemocytes stimulated for 1h with Ringer’s, stained with Hoechst 33342, and visualized with DIC (a, c) and fluorescence microscopy (b, d).

(a, b) Extracellular DNA was released by a granulocyte (arrow). (d) The nucleus of the granulocyte releasing DNA (d, bottom) was irregular in shape and showed diffuse staining compared to an adjacent granulocyte (d, top). Extracellular DNA was visible by DIC microscopy as fibrillar structures.


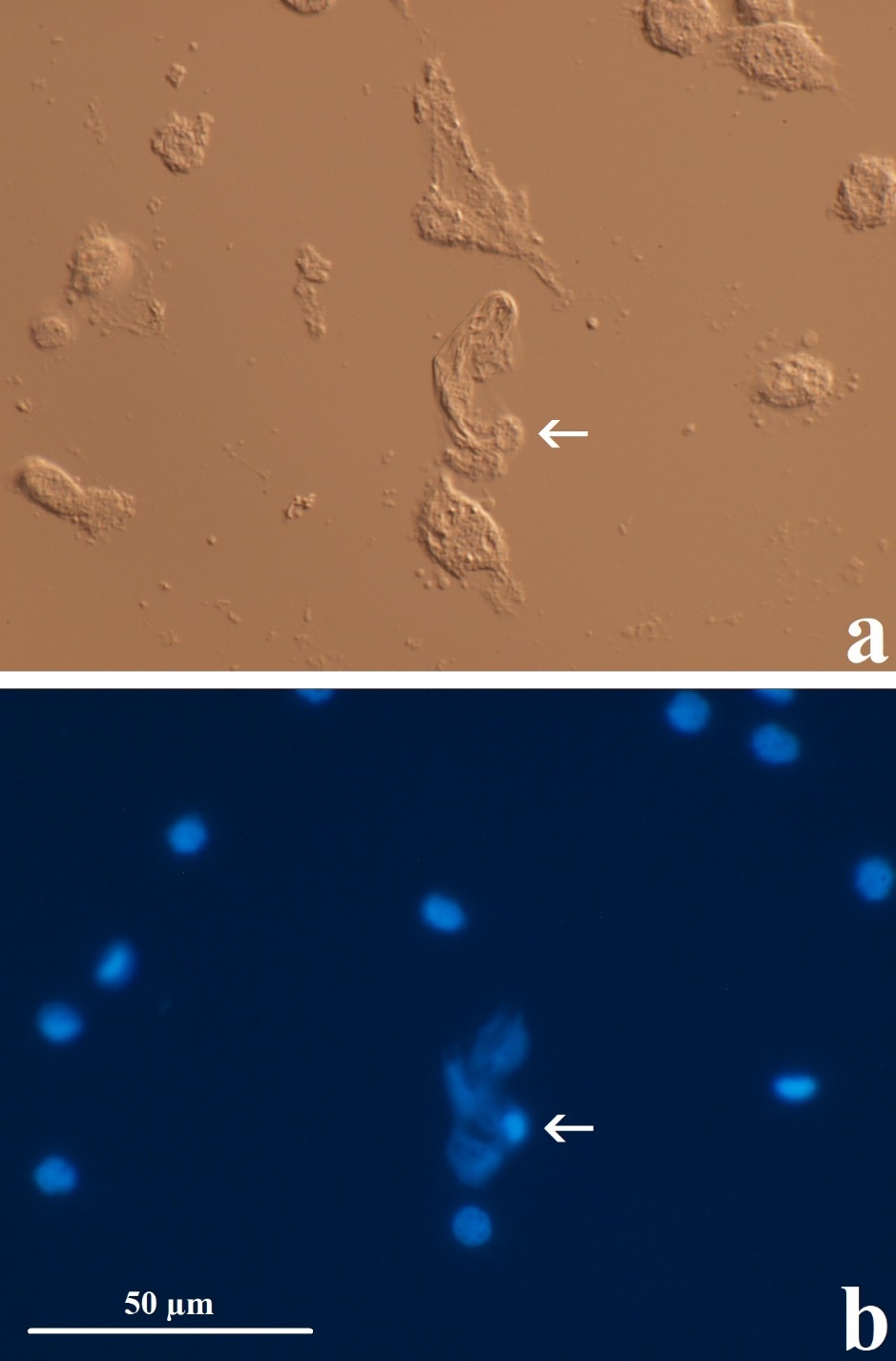


Figure S.2 *G. mellonella* hemocytes stimulated for 1h with PMA (50 µM), stained with Hoechst 33342, and visualized with DIC (a) and fluorescence microscopy (b).

Extracellular DNA was released by a lysed hemocyte.


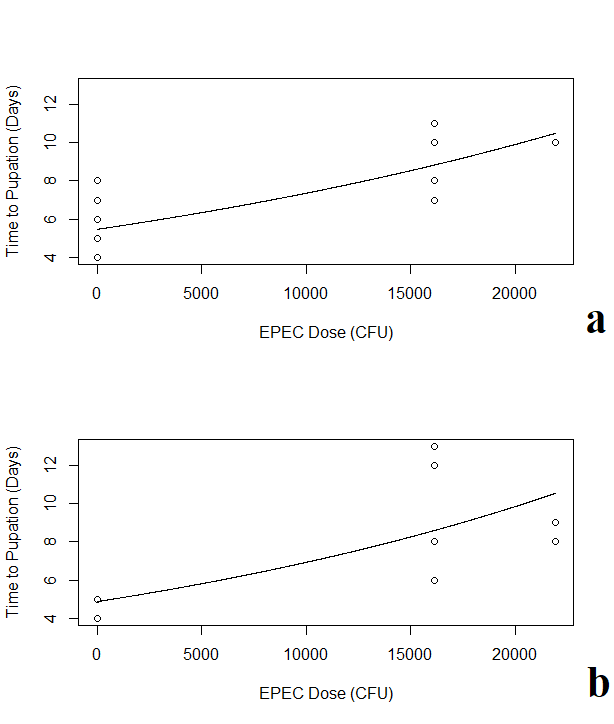


Figure S.3 Time to pupation of *G. mellonella* larvae injected intrahemocoelically with three doses of EPEC (0 CFU Ringer’s control, 1.6 × 10^4^ CFU, or 2.2 × 10^4^ CFU) (n = 80). (a) Larvae injected with EPEC only and (b) larvae injected with EPEC and 500 ng of DNA did not significantly differ. Time to pupation increased as EPEC dose increased.


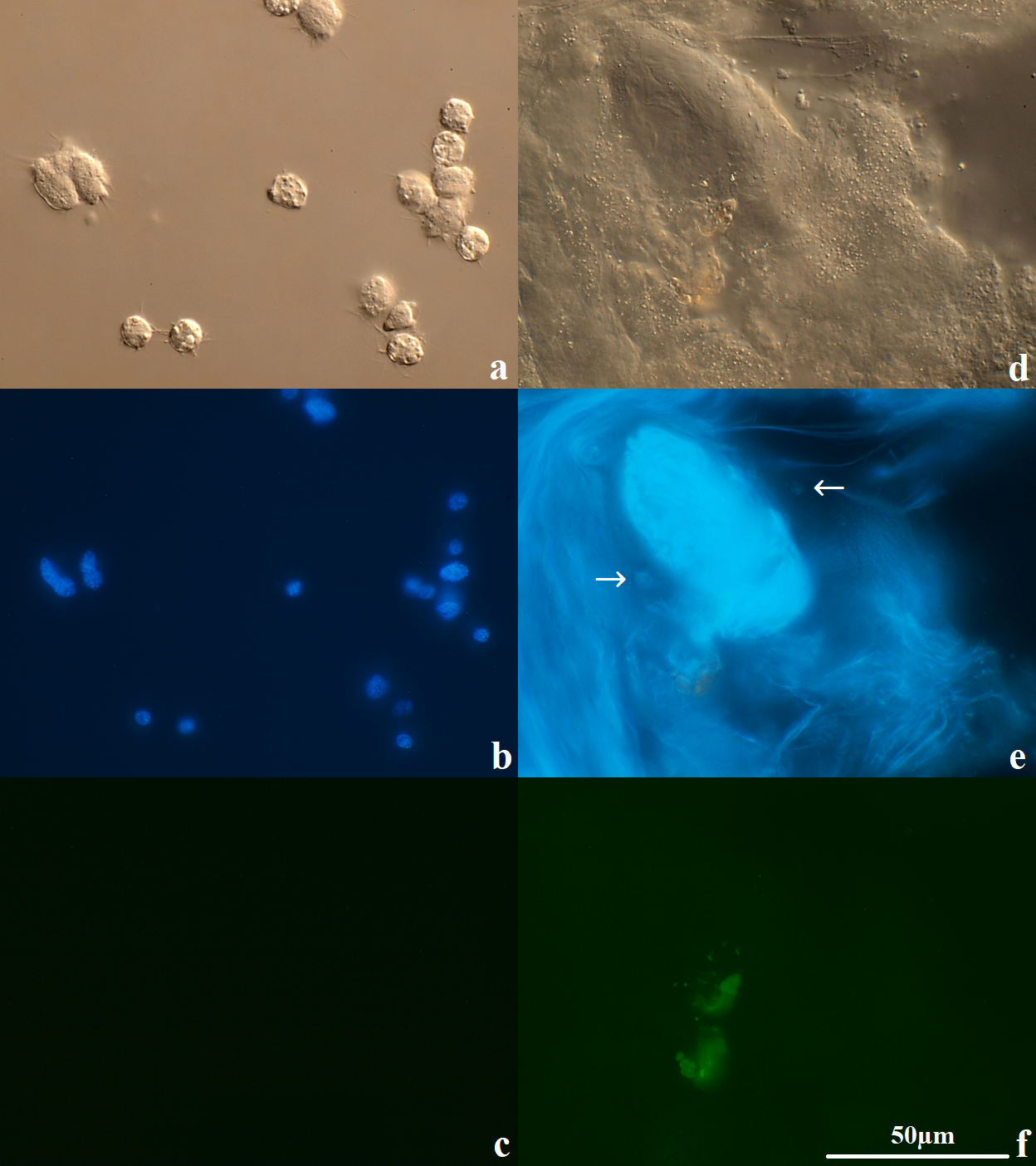


Figure S.4 Melanized coagulated mass from the hemocoel of a live *B. mori* larva 24h after intrahemocoelic injection of EPEC (2.0×10^6^ CFU, n = 8) (right column) and hemolymph of a control insect injected with Ringer’s (n = 5) (left column). (a - c) Control insects did not result in the formation of coagulated masses and displayed no melanization, extracellular DNA, or green fluorescence in the hemolymph. (d and e) Hemocytes (arrows) are embedded in the mass and may be contributing to the extracellular DNA in this mass. (e and f) Extracellular DNA can be seen co-localized with the GFP-expressing EPEC. Extracellular traps were found in 2 out of 8 insects injected with EPEC. The images were captured at 500x magnification using DIC and fluorescence microscopy (for Hoechst and GFP) at the same field of view and focal plane.


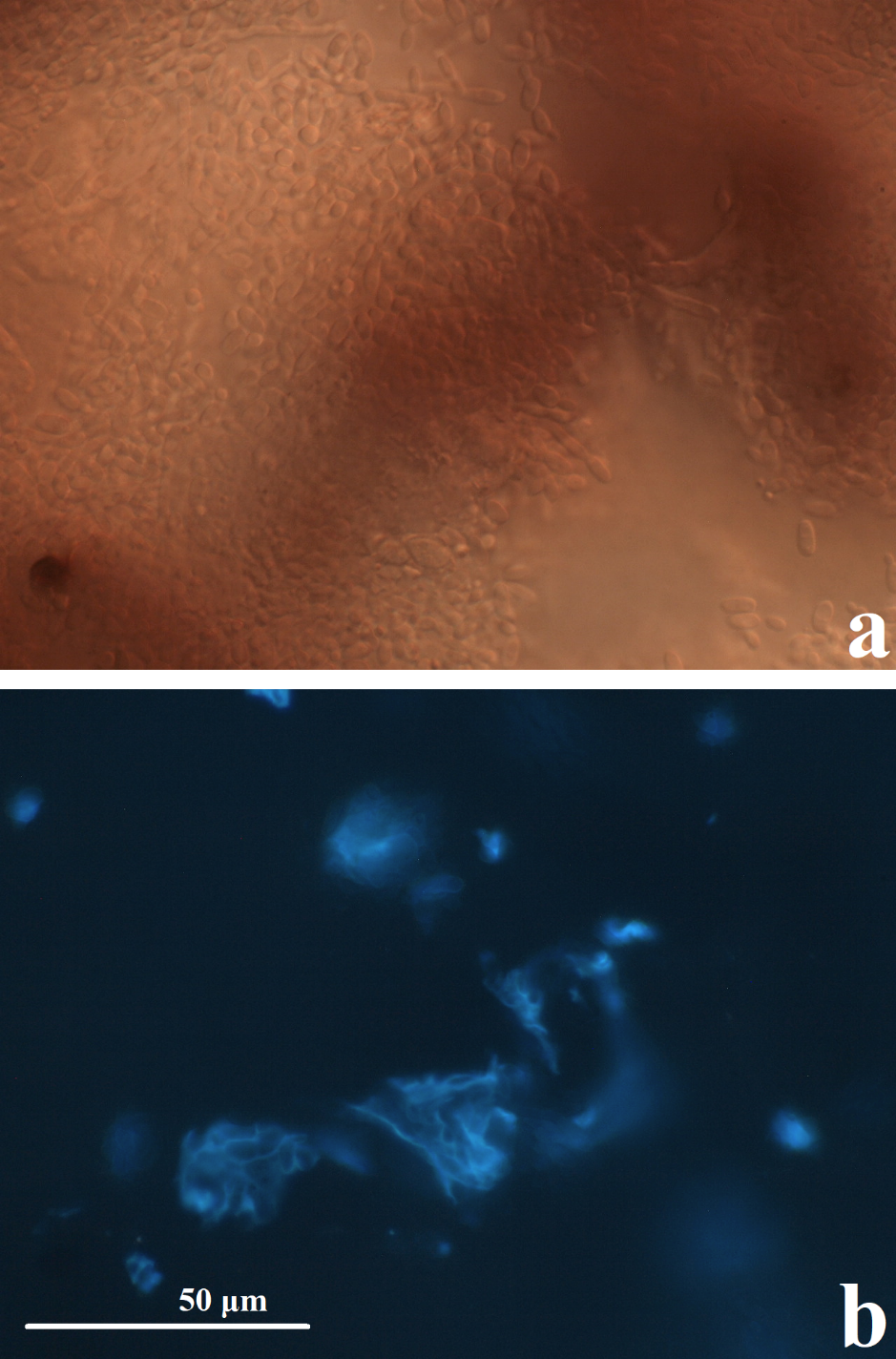


Figure S.5 Melanized coagulum from a *G. mellonella* larva at 24h after intrahemocoelic injection of approximately 10^4^ *C. rugosa*. The sample was stained with Hoechst 33342, and visualized with DIC (a) and fluorescence microscopy (b).

Extracellular DNA (blue fluorescence) was found within the coagulum that trapped numerous yeast cells.


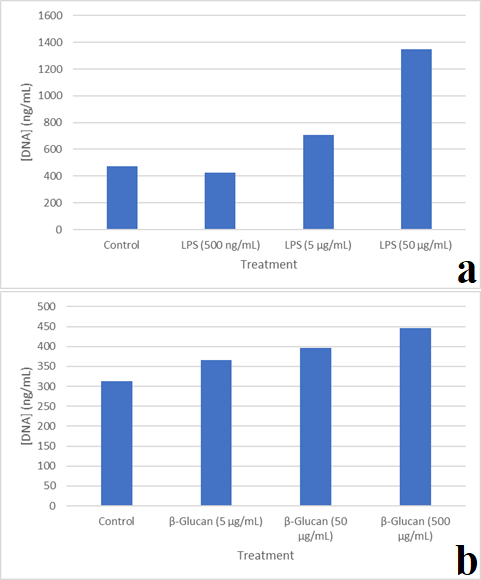


Figure S.6 Extracellular DNA released by *G. mellonella* hemocytes stimulated for 1h with LPS (a) or β-glucan (b) in a preliminary experiment. Insect Ringer’s solution used to dissolve or suspend LPS or β-glucan was used as control. Hemocytes (3.19 × 10^6^ and 2.93 × 10^6^ for a and b respectively) were collected, washed, and resuspended in Grace’s insect medium as described in section 2.3 of this manuscript. Hemocytes were allowed to adhere to the inner surface of a sterile 2 mL microfuge tube for 30 min before stimulation. Supernatant was collected by centrifugation at 200g for 10 min. Extracellular DNA concentration in the supernatant was quantified by Qubit fluorometer. Both LPS and β-glucan induced dose-dependent extracellular DNA release from *G. mellonella* hemocytes.
