# Supplementary material for: Galleria mellonella (Lepidoptera: Pyralidae) Hemocytes Release Extracellular Traps That Confer Protection Against Bacterial Infection in the Hemocoel
Source: J Insect Sci. 2021 Dec 4;21(6):17. doi: 10.1093/jisesa/ieab092 (PMC8643984; doi:10.1093/jisesa/ieab092)
Supplement: ieab092_suppl_Supplementary_Appendices [file ieab092_suppl_supplementary_appendices.docx]

# Appendix A: *G. mellonella* artificial diet

| **Diet ingredient:** | **% by mass:** |
| --- | --- |
| Wheat germ | 28.2 |
| Brewer’s yeast | 14.1 |
| Beeswax (Shredded) | 22.4 |
| Glycerol (Sigma-Aldrich) | 14.1 |
| Honey | 14.1 |
| Water * | 7.1 |
| Glycerol, honey, and water were added to an Erlenmeyer flask and heated (without boiling) on a hotplate until the honey is fully dissolved using a magnetic stir bar. Wheat germ, brewer’s yeast, and beeswax were mixed thoroughly with the solution in a mixing bowl.  * Water used for all artificial diets, media, and solutions in this thesis was purified to approximately 15 MΩ-cm by a research grade ultrafiltration water polishing system (Modulab Model LBPUU 10 1002, Continental Water Systems). | |

# Appendix B: Solutions and media

## **Appendix B.1 Insect Ringer’s solution**

Modified from Ephrussi & Beadle (1936)

| **Component:** | **Concentration (mM):** |
| --- | --- |
| NaCl (Fisher) | 128 (for *B. mori*) or  191.6 (for *G. mellonella*) * |
| CaCl_2_ (Fisher) | 18 |
| KCl (Fisher) | 1.3 |
| NaHCO_3_·2H_2_O (Sigma-Aldrich) | 2.3 |
| pH: 6.0 (Measured)  Osmolality: 314 mOs/kg (Calculated)  Filter-sterilized (0.2 µm)  * Osmolality of Ringer’s solution was adjusted to 440 mOs/kg with NaCl to match *G. mellonella* hemolymph osmolality (Mead *et al.*, 1986). | |

## **Appendix B.2 Anticoagulant antimelanization solution**

Modified from Haine *et al.* (2007)

| **Component:** | **Concentration (mM):** |
| --- | --- |
| NaOH (Merck) | 98 |
| NaCl (Fisher) | 145 |
| Ethylenediaminetetraacetic acid (EDTA) (Fisher) | 17 |
| Citric acid (Fisher) | 41 |
| L-Glutathione reduced (Sigma-Aldrich) | 10 |
| pH: 5.0 (Measured)  Osmolality: 444 mOs/kg (Calculated)  Filter-sterilized (0.2 µm) | |

## **Appendix B.3 Luria-Bertani medium**

Modified from Bertani (1951)

| **Component:** | **Mass (g/500 mL medium):** |
| --- | --- |
| Bacto tryptone (BD) | 5 |
| Bacto yeast extract (BD) | 2.5 |
| NaCl (Fisher) | 5 |
| Bacto agar (BD) * | 7.5 |
| Water | 475 |
| pH: 6.6 (Measured)  Sterilized by autoclaving (Liquid cycle, 15 minutes)  Final volume was adjusted to 500 mL  * Bacto agar was used to make LB agar plates. | |

## **Appendix B.4 Grace’s insect medium**

Modified from Grace (1962)

| **Category:** | **Component:** | **Mass (mg/L medium):** |
| --- | --- | --- |
| Salts | KCl (Fisher) | 4100 |
|  | CaCl_2_ · 2H_2_O (Sigma-Aldrich) | 1320 |
|  | MgCl_2_ · 6H_2_O (BDH) | 2280 |
|  | MgSO_4_ (BDH) | 1360 |
|  | NaH_2_PO_4_ (Sigma-Aldrich) | 866 |
|  | NaHCO_3_ (Fisher) | 350 |
| Soluble amino acids  (Sigma-Aldrich) | L-Arginine HCl | 700 |
|  | L-Aspartic Acid | 350 |
|  | L-Asparagine | 350 |
|  | L-Alanine | 225 |
|  | B-Alanine | 200 |
|  | L-Glutamic Acid | 600 |
|  | L-Glutamine | 600 |
|  | Glycine | 650 |
|  | L-Histidine | 2500 |
|  | L-Isoleucine | 50 |
|  | L-Leucine | 75 |
|  | L-Lysine HCl | 625 |
|  | L-Methionine | 50 |
|  | L-Proline | 350 |
|  | L-Phenylalanine | 150 |
|  | DL-Serine | 1100 |
|  | L-Threonine | 175 |
|  | L-Valine | 100 |
| Insoluble amino acids  (Sigma-Aldrich) | 2N HCl (Fisher) | 2 mL |
|  | L-Cystine | 44 |
|  | L-Tryptophan | 200 |
|  | L-Tyrosine | 100 |
| Carbohydrates  (Sigma-Aldrich) | α-Ketoglutaric Acid | 370 |
|  | Fructose | 400 |
|  | Fumaric Acid | 55 |
|  | D-Glucose | 700 |
|  | Malic Acid | 670 |
|  | Succinic Acid | 60 |
|  | Sucrose | 26680 |
| Vitamins  (Sigma-Aldrich) | Thiamine HCl | 0.02 |
|  | Riboflavin | 0.02 |
|  | D-Ca Pantothenic Acid | 0.02 |
|  | Pyridoxine HCl | 0.02 |
|  | P-Aminobenzoic Acid | 0.02 |
|  | Folic Acid | 0.02 |
|  | Nicotinic Acid | 0.02 |
|  | i-Inositol | 0.02 |
|  | Biotin | 0.01 |
|  | Choline Chloride | 0.2 |
| Water | | 927 mL |
| pH: 6.1 (Adjusted with 10N KOH)  Final volume was adjusted to 1L  Filter-sterilized (0.2 µm)  Medium was prepared and provided by G. J. Hilchie (University of Alberta). | | |

## Appendix B.5: Literature cited

Bertani, G., 1951. Studies on lysogenesis. I. The mode of phage liberation by lysogenic *Escherichia coli*. J. Bacteriol. 62, 293–300.

Ephrussi, B., Beadle, G.W., 1936. A Technique of Transplantation for *Drosophila*. Am. Nat. 70, 218–225.

Grace, T.D.C., 1962. Establishment of four strains of cells from insect tissues grown *in vitro*. Nature 195, 788–789. https://doi.org/10.1038/195788a0

Haine, E.R., Rolff, J., Siva-Jothy, M.T., 2007. Functional consequences of blood clotting in insects. Dev. Comp. Immunol. 31, 456–464. https://doi.org/10.1016/j.dci.2006.08.004

Mead, G.P., Ratcliffe, N.A., Renwrantz, L.R., 1986. The separation of insect haemocyte types on Percoll gradients; methodology and problems. J. Insect Physiol. 32, 167–177. https://doi.org/10.1016/0022-1910(86)90137-X

# Appendix C: Experimental design

| **Treatment** | **EPEC dose (CFU)** | **DNA (ng)** | **Larvae injected** | **Larvae bled** |
| --- | --- | --- | --- | --- |
| 1 | 0 | 0 | 60 | 40 |
| 2 | 0 | 500 | 60 | 40 |
| 3 | 1.6 × 10^4^ | 0 | 30 | 20 |
| 4 | 1.6 × 10^4^ | 500 | 30 | 20 |
| 5 | 2.2 × 10^4^ | 0 | 30 | 20 |
| 6 | 2.2 × 10^4^ | 500 | 30 | 20 |
